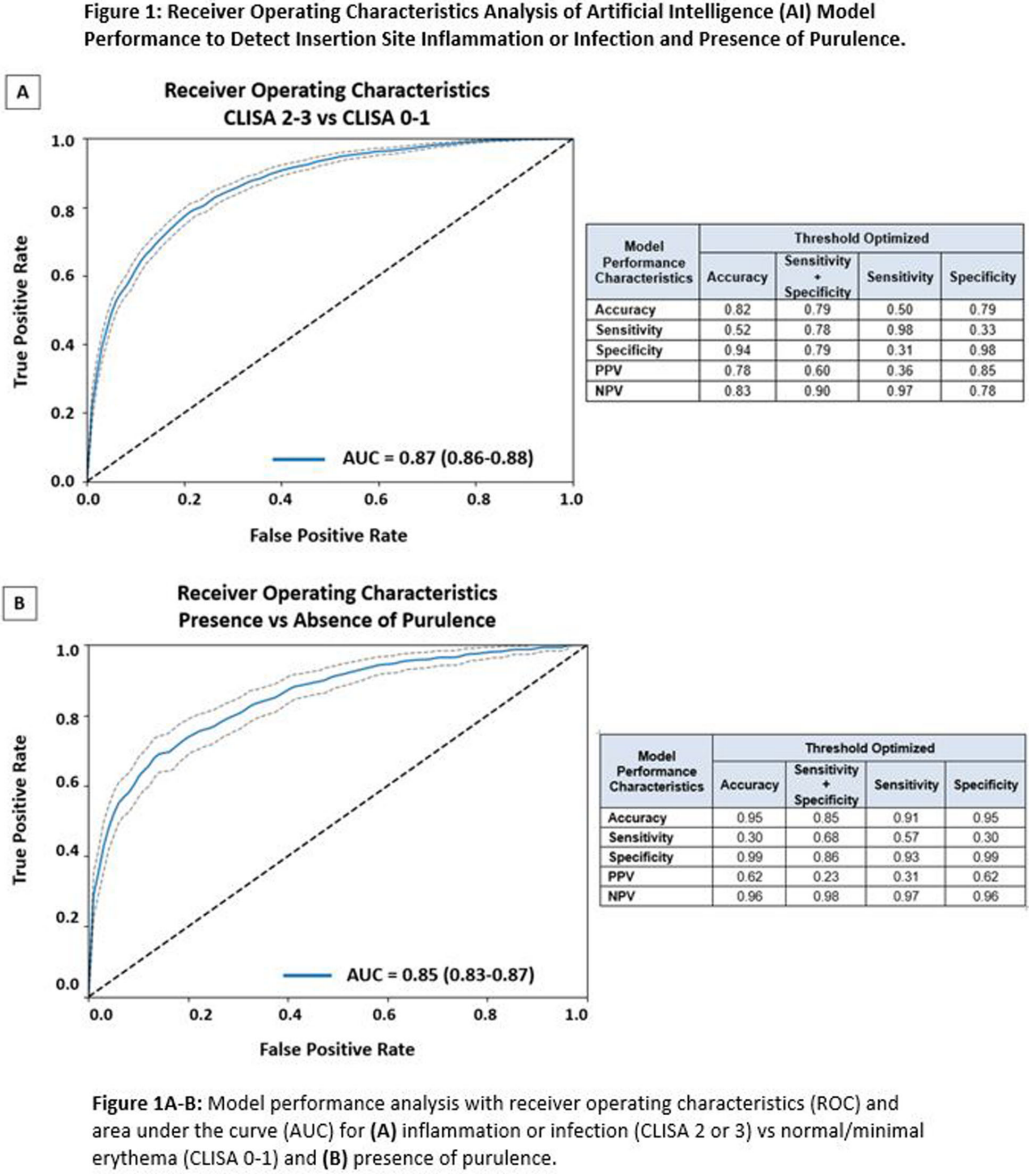# 27 Impact of Inpatient MRSA decolonization strategy at 9 hospital system in Eastern North Carolina

**DOI:** 10.1017/ash.2026.10432

**Published:** 2026-06-23

**Authors:** Shruti Gohil, Amarah Mauricio, Chanon Chantaduly, Joshua Kawaguchi, Justin Ling, Thomas Tjoa, Susan Huang, Peter Chang

**Affiliations:** 1 University of California, Irvine; 2 Division of Infectious Diseases, University of California, Irvine School of Medicine, Irvine, California; 3 University of California, Irvine - Health; 4 UC Irvine; 5 Division of Infectious Diseases, University of California Irvine School of Medicine; 6 University of California Irvine School of Medicine; 7 University of California Irvine

## Abstract

**Background:** Skin changes can precede central line infections by days to weeks, making early identification essential for prevention. Routine nursing assessments help prevent CLABSIs in hospitals, but comparable surveillance is lacking for patients managing central lines at home, leading to preventable infections and readmissions. Hypothesizing that artificial intelligence (AI) can be trained to reliably detect visible inflammation/infection from a photo, we developed algorithms for auto-detection of early signs of localized skin inflammation or infection. **Methods:** We conducted a secondary analysis of photos collected from 3 cohorts of adult patients with central venous catheters (CVCs) at a large academic hospital, an outpatient oncology clinic, and 6 nursing homes between January 2014-June 2017. Skin tone was recorded using the Fitzpatrick Scale, as erythema may be harder to detect in darker skin tones (types IV–VI). Insertion-site inflammation or infection was assessed in each photo using the Central Line Insertion Site Assessment (CLISA) score (Table 1) by trained research staff, with two-physician review. A convolutional neural network model was trained de novo from random weights (binary cross-entropy loss) to predict binarized CLISA score, defined as normal/low risk (non-actionable) scores of 0 or 1 vs high risk (actionable) score of 2 or 3. Five-fold cross-validation estimated model performance for accuracy, sensitivity, specificity, positive predictive value (PPV), negative predictive value (NPV), and area under the curve receiver operating characteristic (AUC/ROC) with 95% confidence intervals. **Results:** Among 5,551 photographs of 964 CVCs in 874 adults, there were 1,191 (21%) photos from hospitalized patients, 2,539 (46%) from outpatient oncology patients, and 1,821 (33%) from nursing home residents. Mean (SD) age was 60 (17), 54% were male, and 183 (21%) patients had darker skin tones (Fitzpatrick skin type IV-VI), Table 2. Among all photos, 1,585 (29%) had localized inflammation/infection (CLISA 2 or 3) and 298 (0.1%) had purulence. Compared to gold standard clinician assessment, AI model performance optimized for accuracy was able to distinguish between normal/low risk (CLISA 0-1) and inflamed/infected catheters (CLISA 2 or 3): accuracy 0.82, sensitivity 0.52, specificity 0.94, PPV 0.83, NPV 0.78, AUC 0.87 (95% CI 0.86-0.88); results were similar for models detecting purulence, Figure 1A-B. **Conclusion:** AI algorithms can identify CVCs with photographic signs of inflammation or infection and signal the need for intervention to prevent CLABSI. AI-autodetection of high-risk catheters can extend hospital CLABSI prevention to settings with limited monitoring, using photographs alone and minimizing reliance on patient self-assessment or care escalation.

**Figure:**
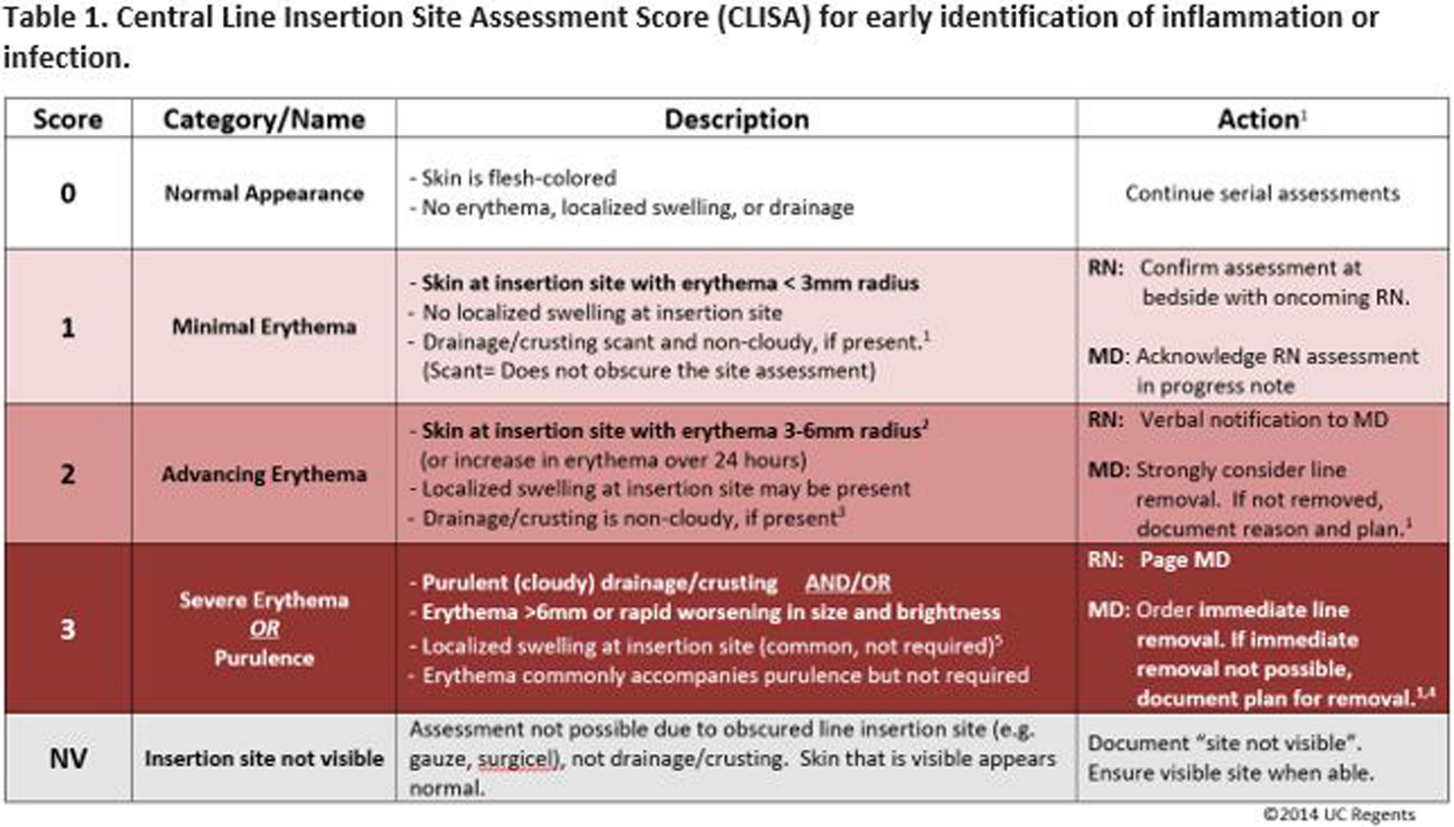

**Figure:**
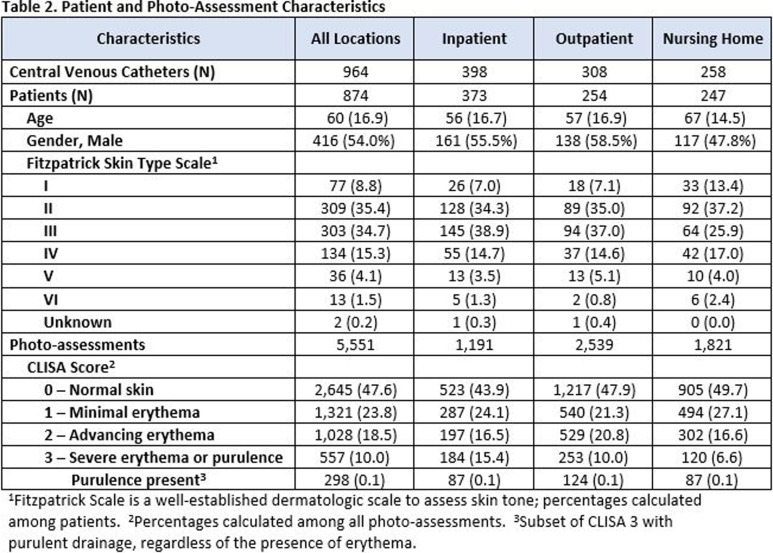

**Figure:**